# High‐Intensity, Parallel Ultrasound Tightening of Facial Skin: Clinical and Pathologic Results

**DOI:** 10.1111/jocd.16670

**Published:** 2024-11-12

**Authors:** Dong Hye Suh, Sang Jun Lee, Kye Yong Song, Hye‐Jin Ahn, Min Kyung Shin

**Affiliations:** ^1^ Arumdaun Nara Dermatologic Clinic Seoul Korea; ^2^ Department of Pathology, College of Medicine Chung‐Ang University Seoul Korea; ^3^ Department of Dermatology Kyung Hee University College of Medicine, Kyung Hee University Hospital Seoul Korea

To the Editor,

Over time, the breakdown of collagen and elastin in the dermis leads to a decrease in elasticity and volume. Clinically, this can contribute to wrinkles, rough texture, and laxity. The demand for noninvasive skin tightening continues to grow [1, 2]. In our study, histological analysis confirmed an increase in collagen and elastic fiber especially in the mid dermis after high‐intensity, parallel ultrasound (HIPS) tightening (Sofwave, SofWave Medical Ltd., Israel).

HIPS tightening is an innovative treatment method for skin laxity, generating thermal effects at depths of 1–2 mm while preserving the overlying epidermis [1]. A new‐generation ultrasound device, Sofwave, was developed to utilize a Synchronous Ultrasound Parallel Beam Technology (SUPERB Technology, Sofwave, Yokneam, Israel), which uses seven parallel transducers in direct contact with the skin to deliver coagulative energy to the mid dermis [1]. Wang et al. [1] reported that HIPS tightening generates the elongated thermal zones that are oriented parallel to the alignment of collagen fibers. The contraction of collagen forms vector lines that align with the direction of facial wrinkles and lines caused by fragmented and irregularly arranged dermal fibers. The thermal effect induces an inflammatory response, ultimately resulting in collagen remodeling through neocollagenesis and neoelastogenesis [1, 2]. There have been not much literature on the high‐intensity, parallel ultrasound. This study aimed to evaluate the efficacy and safety of HIPS tightening of the face and verify histological differences.

Thirteen female participants aged 23–67 years (mean age: 46.5 years) with Fitzpatrick skin types III (53.8%), IV (38.5%), and V (7.7%) (Table 1) were included, and written informed consent was obtained. Prior to treatment, participants applied a topical anesthetic cream (Lidocaine; DaeHan NewFarm, South Korea) for 1 h, washed their faces, and applied an ultrasound gel. The participants received a single HIPS tightening treatment for the entire face and neck. The pulse energy was 3.0–3.9 J and 2–3 passes were delivered to the treatment area on both cheeks. The total pulse was 150–200 for both cheeks.

**TABLE 1:** Participants' demographic data and subjective and objective 5‐point scores.

No.	Sex	Age (years)	Skin type	Subjective score	Objective score
1	F	52	IV	5	3
2	F	26	III	5	5
3	F	55	IV	5	4
4	F	51	III	3	3
5	F	49	IV	4	4
6	F	37	III	4	4
7	F	56	IV	5	5
8	F	52	III	5	5
9	F	58	III	5	5
10	F	54	III	5	4
11	F	47	IV	4	4
12	F	39	V	5	5
13	F	28	III	4	4

*Note:* Satisfaction scores: 1 = no improvement. 2 = 1%–24% improvement. 3 = 25%–49% improvement. 4 = 50%–74% improvement. 5 = 75%–100% improvement.

Participant satisfaction was recorded as subjective 5‐point scores, and two experienced dermatologists, blinded to the study, evaluated the improvement in nasolabial folds and jaw tightening based on Facial Laxity Rating Scale by Leal Silva [3]. They reviewed photographs of the participants, assessed skin laxity and evaluated the objective 5‐point scores. Skin biopsies were taken from the right malar region of all participants before and 2 months after treatment. Density analysis was performed using the ImageJ software according to the method published by Suh et al. (2020) [4]. For all statistical tests, a *p* value of < 0.05 was considered statistically significant. Data were analyzed using SPSS software (version 23.0; SPSS Inc., Chicago, IL, USA).

Collagen remodeling was confirmed at a depth of 1–3 mm from the epidermis. After 2 months of treatment, the mean collagen fiber density increased in the mid dermis from 0.849 (± standard deviation [SD], 0.54) to 1.432 (±0.86) (*p* = 0.094) (Figure 1A), and alignments of collagen fibers showed organized and tightly packed (Figure 1B). Neoelastogenesis and straightening of elastic fibers were also shown. The mean elastic fiber density increased in the mid‐dermis from 0.504 (±SD 0.34) to 0.673 (±SD 0.34) after 2 months of treatment (*p* = 0.033) (Figure 1C).

**FIGURE 1:**
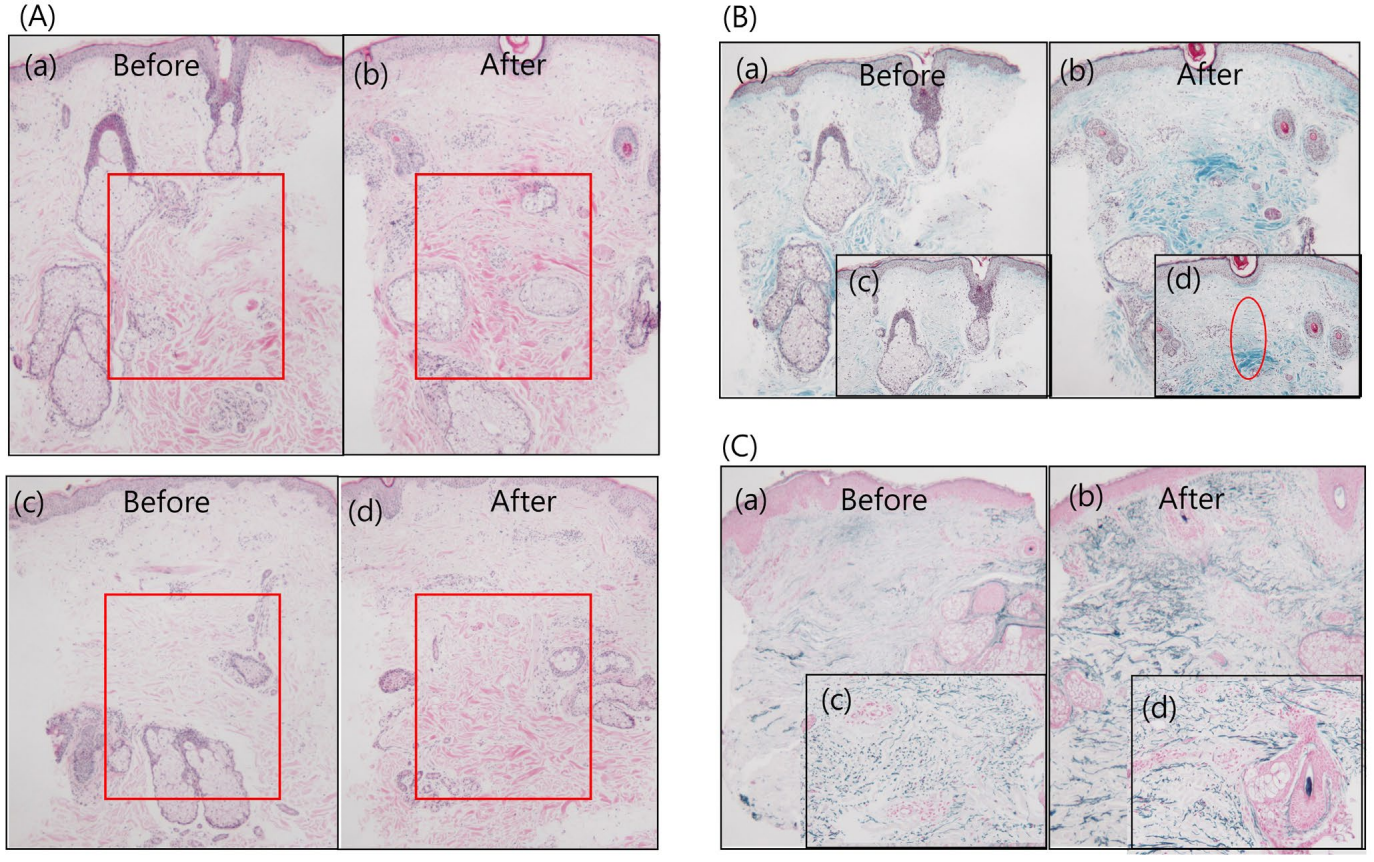
Histologic samples from two participants (A (a), (c)) show increased collagen density at the depth of 1–3 mm (red box) after 2 months (A (b), (d)): Hematoxylin and eosin staining, original magnification, ×40. Increased collagen densities after treatment were confirmed through Masson trichrome staining (B(a), (b)): original magnification ×40, and uniform collagen alignments(red circle) appeared as results of collagen remodeling (B(c), (d)): original magnification ×100. Entire dermal elastic fiber increases distinguished by Victoria blue (VB) staining between before (C(a)) and after (C(b)): original magnification ×40. Long and straightened elastic fibers increased after treatment (C(d)) compared before treatment (C(c)): VB, original magnification ×100.

The subjective 5‐point scores showed that 12 participants (92%) perceived a moderate‐to‐excellent improvement (> 50%) in their condition. According to the objective 5‐point scores, 11 patients (85%) showed moderate‐to‐excellent (> 50%) improvement (Table 1). There were no adverse events such as fat atrophy, persistent erythema, swelling, bruising, or prolonged numbness.

In the young dermis, intact collagen fibrils are abundant, tightly packed, and well organized. In contrast, collagen fibrils are fragmented and disorganized in the aged dermis [5]. These alterations of collagen fibers compromise skin integrity. The aging process also disrupts the elastic fiber network. In intrinsically aged skin, elastic fibers shorten and fragment even without sun exposure [6]. Elastin and elastic fibers are distinctive due to their very low and slow turnover rates. It is unlikely that elastic fibers are appreciably replaced in skin naturally [6]. Thus, replenish collagen and elastin fibers and support the structure of networks is important for rejuvenate the skin. In conclusion, high‐intensity, parallel ultrasound tightening is safe and effective for reducing facial laxity.

## Conflicts of Interest

The authors declare no conflicts of interest.

## Data Availability

The data that support the findings of this study are available from the corresponding author upon reasonable request.
